# The Relations of Dentistry to Medicine, and Aseptic Dentistry

**Published:** 1890-03

**Authors:** J. S. Wight

**Affiliations:** Professor of Operative and Clinical Surgery at the Long Island College Hospital


### International Dental Journal.

Vol. XI. March, 1890. No. 3.

### Original Communications.[1]

### THE RELATIONS OF DENTISTRY TO MEDICINE, AND ASEPTIC DENTISTRY.[2]

BY J. S. WIGHT, M.D.,

Professor of Operative and Clinical Surgery at the Long Island College Hospital.

As a matter of fact and by common consent medicine is a generic term. It covers the entire field in which we work to prevent and cure disease. And it must, in this sense, include the department of surgery as well. And in whatever way we deal with surgery, we consider matters that relate to the question of medicine,—either preventive or curative. Now, it so happens that all of the specialty which we call dentistry occupies, for the most part, the field of surgery; it is a special kind of handwork. But this fact does not exclude another fact. In dentistry there are many questions that relate to medicine as distinguished from surgery. Then, we have this conclusion: Dentistry is both medical and surgical.

Let me say that dentistry is a special practice. It appears to have started in a small way,—as the art of pulling teeth. It has risen step by step, without the suggestion, the aid, and the support

[1] The editor and publishers are not responsible for the views of authors of papers published in this department, nor for any claim to novelty, or otherwise, that may be made by them. No papers will be received for this department that have appeared in any other journal published in this country.

[2] Read before the Brooklyn Dental Society, January 27, 1890.

directly of medicine, until it has been built up into a system of practice, which everywhere confers benefits and improves health. Other specialties have set out from medicine, and have erected themselves into a somewhat independent existence. And the more they keep out of sight the great principles of medicine, the less their beneficent progress. We may admit at once that special medicine relates to special organs; and then we may say that special organs are related to the general organism. Let the general organism suffer, and special organs suffer also. And this means that the specialist who has not general knowledge, and who has special knowledge, from the very nature of the case, has incomplete, imperfect, and insufficient knowledge. That is to say, no man can be a complete specialist who has not acquired a general knowledge of medicine. And I am now going to admit that the dentist comes very near an exception to this rule: a good dentist may exist without a knowledge of general medicine. And yet, permit me to add that, if he had a knowledge of practical medicine, he could accomplish, in the main, better results. Not that I would have dentists become general practitioners,—that is not what I mean. It is this: I would have general medicine throw such light as it could upon the entire field of dentistry. And so I would encourage the dentist to move his camp somewhat over into the field of general medicine.

How shall this relation be arranged? I am of the opinion that the dentist might derive much advantage from the attendance on one course of medical lectures; two courses of lectures would be better still. And if he cannot attend regularly, he would derive much benefit from lectures on anatomy, physiology, general medicine, and the principles of surgery. Ought the dentist to be a doctor? This is not my point. Under no conditions should he practise medicine and surgery,—even though he have a medical degree. My point is this: the dentist could practise the special surgery and the special medicine relating to the teeth and mouth, contained under the head of dentistry, if his special knowledge is supplemented by a reasonable degree of general medical knowledge.

Let us approach this subject from another stand-point. Suppose a man who never studied medicine comes forward and sets himself up as a specialist in diseases of the skin, or the eye, or the ear, or of the uterus, what would be said in regard to the matter? He may be exceptionally clever, in some respects, in what he professes and undertakes; his practice may be followed by a good degree of success; and he may be well educated in many directions. Having not the least intention of throwing any imputation or the least reflection on dentistry, I may say that, by common consent, the dentist does most admirable work from just this stand-point. In fact, he is an eminent specialist, without the general knowledge that should underlie the practice of every specialty.

And then, there is another relation which dentistry bears to medicine. Does my patient have diseased teeth, I cannot well cure him until his teeth are attended to. Well, if I doctor them, I am not sure that everything is all right; he may improve or he may not; but if I send him to the dentist, who knows how to doctor his teeth, then everything goes well; my patient improves, not altogether on account of the doctor for the teeth, but because what I can do for him is not now undone by the irritation of diseased teeth. You will see that I admit the incomplete knowledge of the general practitioner in the field of your specialty; and that you can co-operate with him not only in curative, but also in preventive medicine. It is not necessary for the doctor to know all about dentistry,—and yet he ought to have some general idea about the subject: then he will be a more competent practitioner. Nor is it necessary for the dentist to know all about medicine, but he would be able to do better work if he had a good general knowledge of its principles. Our meaning is not that the dentist should practise medicine, nor that the doctor should practise dentistry,—still there may be an exception to this rule. In some of the rural districts, it might be important for the doctor to know something about dentistry. He may know how to extract teeth in a scientific way; he may know how to keep teeth clean; and he may have some idea as to the necessity of saving and filling teeth: then he can advise his patients to go to a dentist, who may be found not many miles away. In fact, this point has been considered by some of the medical colleges; and so there is a tendency towards the establishment of a dental specialty in the curriculum of medical teaching,—much the same as we have other specialties.

In the next place, a subject which has a great interest for me—and I doubt not for others—comes to my mind for consideration,—that is, the subject of *aseptic dentistry*. It is true that dentistry is more in the line of surgery than of medicine; and if we approach it from this side, we shall obtain clearer ideas about it. And then let us set out with facts that will illustrate our subject. When I speak of infection, I mean the same thing as blood-poisoning. And it is about certain facts relating to infection that I am going to speak. A very high death-rate occurred at one time in the College

Hospital from septic fever among the surgical patients. On the supposition that the infection was local, the beds and the wards were cleaned in the following way: They were steamed for twenty-four hours with the vapor of carbolic acid and vinegar; the walls were washed with a carbolic acid solution; the clothing was immersed in boiling water; and then the wards were thoroughly ventilated. The patients were put into the disinfected ward, and sepsis went on much the same as before. Evidently the difficulty had not been reached, nor had the fatal influence been removed. Had anything been overlooked in the work of disinfection,—that is, on the ground that there is such a thing as infection? I found that my own hands had not been duly considered, nor those of my assistants. And then I asked myself if I had used the best disinfectant? My answer to this question was that I had not. Now, let me tell you what I did. Understand that I was impressed with the importance of keeping my patients from dying. They were surgical cases. Then I selected chlorine, as it is found in chlorinated soda. After washing off the ordinary dirt, I applied chlorinated soda to my hands, sometimes full strength and sometimes diluted. You know the surgeon operates with his hands, and they come in contact with the fresh surfaces; my hands were thus made surgically clean, so they could not infect the wounds I made with my knife. And what was the result of this change in practice? As time went on, more and more infection and sepsis diminished, while at the outset a very marked change took place for the better. There were more speedy recoveries, and fewer deaths occurred. And what was the meaning of this? Simply we had demonstrated that the surgeon, in trying to save life, had carried the causes of death on the very hands he put forth to rescue and save. Here were strange secrets that had defied investigation for centuries. A new line of practice had been instituted in the field of preventive medicine,—for such it was,—even though explored by the surgeon. And so what we mean by surgical cleanliness is to disinfect all things that come in contact with our wounds, to prevent them from becoming infected, to permit them to heal in the normal way; that is, to keep the wounds we make aseptic; in other words, to practise antiseptic surgery. So much for our illustration.

Should dentists have clean hands? Should they have clean instruments? When one thinks about it at all, there can be only one way to answer these questions; indeed, they are of primary importance. You will permit me to analyze the practical relations. In the first place, there are two kinds of cleanliness: one ordinary,—that is, the hands are washed clean, so that the eye cannot detect any dirt on them. This condition does not include that of surgical cleanliness, in which the hands have been completely disinfected, when they are harmless, not having any infecting material on them. That is, as we have already said, surgical cleanliness consists in having all infection removed from the hands, in so far as they are concerned. As surgery is handwork, it is very true that dentistry is pre-eminently such kind of work, for it is done with the hand. And the hand should not only be trained, but it should be clean,—I mean the hand of the dentist. And if this is true of the dentist's hand, it is also true of his instruments,—that is, they should be clean; they should not have any infecting material on them. Each instrument should be above suspicion. There should be a reasonable warranty that it is really surgically clean. This is of much more importance than its general appearance. Infecting material is not seen by the eye, but it is none the less potential in all manner of surgical mischief. It shows itself by its works, and its works are for the most part bad.

We will suppose that the hands and instruments of the dentist are aseptic, for that is what surgical cleanliness really means. He begins his professional work for the day, then, when he has made his hands and instruments aseptic. Well, he extracts a tooth; he cleans a tooth; or he fills a tooth. This is all right for the first patient. It may be a patient who would be greatly offended if it were said that his or her mouth is not surgically clean. And yet, in the light of my argument, who would want this dentist to put his instruments into his mouth—not to mention his fingers—just as they come from this first patient's mouth? Is there a dentist here to-night who would like to have such an instrument used in his mouth, or who would use it for one of his patients, before it had been made aseptic again?

Does the dentist know the fearful responsibility he incurs in regard to this question? Let me illustrate. He extracts a tooth from a mouth in which there are specific ulcers; the jaw of the instrument becomes covered with infecting pus; he wipes it off, or he thinks he does; the instrument is ready, like the lancet, for vaccination; an innocent lady takes a seat in his dental chair, and he extracts one of her teeth with this same instrument. It is not my object to-night to trace and picture the long series of unfortunate consequences that may follow. Only let me say, in the name of science, humanity, and charity, that these consequences are entirely avoidable. If this is so, may we suggest where the responsibility rests? Whose fault is it? Any one can answer this question. But the dentist replies, Am I to clean my forceps one hundred times to meet this one possible emergency? Undoubtedly. Which is better, to wash and clean your forceps so many times, or to incur the responsibility of causing so much suffering to an innocent patient who has confidence in your care and skill? Which will give you more trouble, to take a little time to apply the principles of aseptic dentistry, or to meet the legal consequences of ordinary care and skill in your work? For that is indeed what it amounts to in the light of modern science and recent practice.

Let me further illustrate. Science has shown us that consumption—that is tuberculosis—is an infectious disease; that it can be implanted in a perfectly healthy individual; that it can be conveyed from one person to another, and that it is caused by a micro-organism, which is present in the system of those who have it. This disease attacks the lungs frequently. Expectoration follows. The bacilli are in the sputa. And they may lodge in decayed teeth, on the gums, and in other parts of the mouth. Has it occurred to any one present that these micro-organisms can be conveyed from the mouth of a consumptive to the mouth of a healthy person, and cause infection and all the consequences that result from this dread disease? Is any one sure that he has never conveyed the virus of tuberculosis from one person to another by means of instruments that were unclean? We may allow that, hitherto, this effect, which we can hardly doubt, has only been from excusable ignorance; yet, in the future, who can doubt that its repetition must be set down to the account of criminal negligence. And so, what man among you, now knowing what might occur, would hesitate to take thought of this matter, and make his instruments aseptic, as well as his hands, after he has served one patient, and before he comes to serve another?

And may I suggest, from analogy, the possibility of Rigg's disease belonging to the same general subject of infection? The insidious and progressive nature of this disease, extending from point to point, indicates phases of resemblance to other infectious diseases. One cannot explain this disease quite so well on any other theory; and yet this does not prove that Rigg's disease is infectious,—the theory of infection gives us the most reasonable explanation. Suppose this view to be the true one; then, what we have said in regard to syphilis and tuberculosis will indeed be true in regard to Rigg's disease. More than once have I asked myself, Can it be possible that this fearful scourge of the human teeth is infectious? and can it be communicated from one person to another? This question will have to be asked; it will have to be the incentive to investigation; and the answer will be of moment whatever it may be. If the disease is infectious, all may find out a cure; if it is the result of senile changes, we may only be able to palliate it. At all events, there is no reasonable doubt as to the need of having clean instruments,—in other words, it will be good practice to act as if this view were true.

The extent of the field beyond these illustrations is at present unknown,—that is, we do not now know how many pathogenic micro-organisms may be likely to affect the mouth, in one way or another, by means of dental instruments. Let it suffice that we have directed the attention to this important subject; a subject in which lies immense possibilities of harm and peril; a subject which belongs to the field of preventive medicine, in which we find the greatest advancements and the noblest benefactions.

I will try to tell you how to keep your hands and instruments clean; by this, I mean surgically clean,—that is, aseptic. In the first place, the hands should be scrubbed with soap and water, using a large nail-brush. At the same time the nails must be kept thoroughly clean. Then they should be washed with a solution (1 to 5000) of mercuric chloride. Subsequently let them be washed in alcohol from time to time. Or, make a solution as follows: First, mix glycerin and carbolic acid, equal parts; and then make a one-per-cent. solution of this mixture in water, and have it ready for bathing the hands, when required. Other methods need not now be described, since these are sufficient. In the second place, it would be very easy for the dentist to have a small vessel containing water over a gas-jet, burning so as to keep the water at a boiling temperature; in this boiling water the instruments can be immersed after being used in the mouth of one patient, and before being used in the mouth of another. This would insure the destruction of pathogenic micro-organisms, and prevent infection. A plan so simple, and so easy of execution, can be adopted by any operator. One now submits to the true teaching of science; one puts to the best use the principles of asepsis for the benefit of those who suffer; one may not neglect to apply new facts, and so permit harm to come to others who depend on him for aid, and pay him liberally for his assistance. In fine, the law of asepsis rules every part of the great territory of antiseptic work, and in no department more than in dentistry.

